# Deep vein thrombosis as a public health priority: wHO’s agenda for Africa 2030

**DOI:** 10.3389/fcvm.2026.1841390

**Published:** 2026-06-24

**Authors:** Emmanuel Ifeanyi Obeagu

**Affiliations:** 1 Division of Haematology, Department of Biomedical and Laboratory Science, Africa University, Mutare, Zimbabwe; 2 Department of Molecular Medicine and Haematology, School of Pathology, Faculty of Health Sciences, University of the Witwatersrand, Johannesburg, South Africa

**Keywords:** 2030 health goals, Africa, deep vein thrombosis, public health, WHO agenda

## Abstract

Deep vein thrombosis (DVT) is a serious vascular condition characterized by the formation of blood clots in deep veins, predominantly in the lower limbs. Despite its significant morbidity and mortality worldwide, DVT remains an under-recognized public health issue in Africa, where limited awareness, diagnostic challenges, and resource constraints contribute to suboptimal prevention and management. The rising burden of risk factors such as obesity, HIV infection, infectious diseases, and sedentary lifestyles highlights the urgent need to prioritize DVT within the continent's healthcare agenda. The World Health Organization (WHO) has identified the control of DVT as a critical component of its 2030 health strategy for Africa, emphasizing integration into non-communicable disease and cardiovascular health programs. Strategic priorities include enhancing epidemiological surveillance, increasing healthcare provider and community awareness, strengthening diagnostic capacity, and implementing evidence-based thromboprophylaxis guidelines. However, challenges such as inadequate infrastructure, limited access to anticoagulant therapies, and sociocultural barriers hinder effective DVT control across many African settings. Addressing these challenges requires a coordinated, multisectoral approach that incorporates capacity building, resource mobilization, and policy reforms aligned with WHO's vision. By elevating DVT as a public health priority, African nations can improve early detection, prevention, and management of venous thromboembolism, ultimately reducing preventable complications and improving vascular health outcomes across the continent.

## Introduction

Deep vein thrombosis (DVT), defined as the formation of thrombi within the deep venous circulation, most commonly affecting the lower extremities, represents a major contributor to global cardiovascular morbidity and mortality. Together with pulmonary embolism (PE), DVT constitutes the clinical spectrum of venous thromboembolism (VTE), a condition responsible for substantial healthcare burden, long-term disability, recurrent hospitalization, and preventable death worldwide. Although historically perceived as a disease more prevalent in high-income countries, accumulating evidence indicates that DVT is an increasingly important public health challenge in low- and middle-income regions, particularly in Africa, where demographic, epidemiological, and health system transitions are reshaping disease patterns across the continent (1, 2). Globally, venous thromboembolism is recognized as one of the leading causes of cardiovascular mortality after myocardial infarction and stroke. The condition is associated with serious acute and chronic complications including pulmonary embolism, recurrent thrombosis, post-thrombotic syndrome, chronic venous insufficiency, chronic thromboembolic pulmonary hypertension, and reduced quality of life. Importantly, many VTE-related deaths remain preventable through early risk assessment, timely diagnosis, thromboprophylaxis, and evidence-based anticoagulation therapy. Despite these advances, substantial disparities persist between high-income and resource-limited settings regarding access to thrombosis prevention and treatment services (3, 4).

Africa is currently undergoing a significant epidemiological transition characterized by the dual burden of communicable and non-communicable diseases. Rapid urbanization, population growth, aging demographics, sedentary lifestyles, obesity, hypertension, diabetes mellitus, trauma, surgical interventions, and increasing cancer prevalence have collectively contributed to a growing incidence of cardiovascular and thrombotic disorders. Simultaneously, persistent infectious diseases such as HIV/AIDS, tuberculosis, malaria, and emerging viral infections continue to exert profound effects on vascular inflammation, endothelial dysfunction, and coagulation pathways, thereby increasing thrombosis risk among African populations (5). Historically, DVT was considered uncommon among African populations due to limited clinical recognition and underreporting. However, recent studies from tertiary hospitals and regional registries increasingly demonstrate that venous thromboembolism is far more prevalent than previously assumed. Hospital-associated thrombosis, particularly among surgical patients, trauma patients, pregnant women, critically ill individuals, and patients with malignancies or infectious diseases, has emerged as a significant cause of morbidity and mortality across many African healthcare systems. Unfortunately, the true burden of DVT remains underestimated due to inadequate surveillance systems, limited diagnostic capacity, and low awareness among healthcare providers and the general public (6).

The pathophysiology of DVT reflects a complex interaction between venous stasis, endothelial injury, and hypercoagulability, collectively described by Virchow's triad. In African settings, additional disease-specific and socioeconomic factors further modify thrombosis risk. HIV-associated chronic inflammation, tuberculosis-related hypercoagulability, prolonged hospitalization, maternal complications, limited mobility following trauma, and delayed healthcare access all contribute to increased vulnerability to venous thrombosis. Moreover, the expanding burden of cancer and increasing use of invasive medical and surgical procedures have intensified thrombotic risk within healthcare institutions (7). The public health implications of DVT in Africa are substantial. Beyond its direct clinical consequences, DVT contributes to prolonged hospitalization, disability, productivity loss, financial hardship, and increased healthcare expenditure. Long-term complications such as post-thrombotic syndrome may significantly impair mobility, occupational functioning, and quality of life, particularly in settings with limited rehabilitation and chronic care services. In many low-resource environments, out-of-pocket healthcare expenditures associated with diagnostic imaging, anticoagulant therapy, laboratory monitoring, and long-term follow-up create additional socioeconomic burdens for affected individuals and families (8).

The World Health Organization (WHO) Agenda for Africa 2030 and the broader Sustainable Development Goals (SDGs) provide an important strategic framework for addressing emerging cardiovascular and thrombotic diseases across the continent. WHO priorities including universal health coverage, reduction of premature mortality from non-communicable diseases, maternal health improvement, strengthening of surgical safety systems, emergency preparedness, and health systems resilience are directly relevant to thrombosis prevention and care. Although DVT has often received less policy attention than other cardiovascular disorders such as ischemic heart disease and stroke, its integration into broader public health strategies is essential for reducing preventable mortality and improving health outcomes (9, 10). Particularly important is the integration of DVT prevention into hospital care systems. International evidence consistently demonstrates that hospital-associated venous thromboembolism represents one of the leading causes of preventable inpatient death. Implementation of routine thrombosis risk assessment, evidence-based thromboprophylaxis protocols, early mobilization strategies, and standardized anticoagulation practices can substantially reduce morbidity and mortality. However, adoption of these interventions remains inconsistent in many African healthcare institutions due to resource limitations and lack of institutional protocols (7).

Recent advances in thrombosis research have also emphasized the importance of prognostic stratification and individualized management approaches. Baseline clinical and laboratory variables including anemia, renal dysfunction, inflammatory markers, active malignancy, and infectious comorbidities have demonstrated prognostic relevance in predicting VTE outcomes. These findings are especially important in African healthcare environments where access to advanced diagnostic technologies may be limited and cost-effective prognostic tools are urgently needed (11). Furthermore, the COVID-19 pandemic reinforced global recognition of thromboinflammatory disorders and exposed vulnerabilities in healthcare systems regarding thrombosis prevention and management. SARS-CoV-2 infection was strongly associated with endothelial injury, hypercoagulability, and increased incidence of venous thromboembolic complications, particularly among critically ill patients. The pandemic highlighted the necessity of strengthening thrombosis surveillance, anticoagulation protocols, and critical care preparedness within African health systems (12, 13).

Addressing DVT as a public health priority requires coordinated multisectoral action involving policymakers, clinicians, researchers, public health institutions, and international organizations. Improved epidemiological surveillance, healthcare financing, workforce development, public awareness, diagnostic infrastructure, affordable access to anticoagulants, and implementation of evidence-based thromboprophylaxis programs are essential components of an effective continental response (14, 15). This narrative review therefore examines deep vein thrombosis as an emerging public health priority within the framework of the WHO Agenda for Africa 2030. The review explores the epidemiology, pathophysiology, risk factors, clinical outcomes, diagnostic challenges, health system limitations, socioeconomic implications, and prevention strategies associated with DVT in Africa. It further evaluates opportunities for integrating thrombosis prevention and management into broader healthcare strengthening initiatives aimed at achieving universal health coverage and reducing preventable cardiovascular mortality across the continent by 2030.

## Aim

This review aims to highlight deep vein thrombosis (DVT) as a significant but under-recognized public health challenge in Africa, examining the World Health Organization's (WHO) strategic priorities for DVT prevention, diagnosis, and management within the framework of the 2030 health agenda. It seeks to raise awareness among policymakers, healthcare providers, and researchers about integrating DVT interventions into broader cardiovascular and non-communicable disease (NCD) programs.

## Epidemiology and burden in Africa

Deep vein thrombosis (DVT) is an important component of venous thromboembolism (VTE), which includes pulmonary embolism (PE), and together they constitute a significant cause of morbidity and mortality worldwide. However, the epidemiology of DVT in Africa remains poorly characterized, largely due to underdiagnosis, limited healthcare infrastructure, and a paucity of population-based studies. Despite these gaps, available data suggest a rising burden of DVT across the continent, compounded by the increasing prevalence of both traditional and region-specific risk factors (16). Estimates of DVT incidence in Africa vary widely but generally indicate rates comparable to or higher than those reported in high-income countries, particularly in hospitalized and high-risk populations. Studies conducted in urban and tertiary care settings reveal that DVT complicates up to 9%-10% of hospitalized patients, especially those undergoing surgery, prolonged immobilization, or childbirth. However, rural and community-level data remain sparse, limiting understanding of the true population-level burden. The lack of national registries or systematic surveillance contributes to this underestimation (17, 18).

The burden of DVT in Africa is exacerbated by the high prevalence of infectious diseases, notably HIV/AIDS, tuberculosis, and malaria, which induce prothrombotic states through chronic inflammation, endothelial dysfunction, and immune activation. HIV-positive individuals have a two- to ten-fold increased risk of developing DVT compared to HIV-negative populations. Furthermore, antiretroviral therapy and opportunistic infections add complexity to thrombosis risk and management. In addition, conditions such as sickle cell disease, endemic to sub-Saharan Africa, also predispose individuals to venous thrombosis through hemolysis-related endothelial injury and hypercoagulability (19, 20). Non-communicable disease (NCD) risk factors, including obesity, hypertension, diabetes, and sedentary lifestyles, are emerging rapidly due to urbanization and lifestyle changes. These factors are well-established contributors to venous thrombosis globally and are expected to drive increased DVT incidence in Africa. Additionally, increasing rates of surgical interventions, cesarean sections, and cancer diagnoses introduce further thrombosis risk. However, inconsistent application of thromboprophylaxis protocols in healthcare settings leaves many patients vulnerable (21). DVT is associated with considerable morbidity and mortality in Africa. Pulmonary embolism, a potentially fatal complication, often goes undetected or is misdiagnosed due to limited diagnostic capacity, resulting in high in-hospital mortality rates. Post-thrombotic syndrome, characterized by chronic pain, swelling, and venous ulcers, significantly impairs quality of life and productivity but remains largely undocumented in African populations. The economic burden of DVT-related complications is substantial, further straining already resource-limited health systems (22).

## Risk factors unique to Africa

Deep vein thrombosis (DVT) in Africa is influenced not only by the classical risk factors recognized globally but also by several unique regional and context-specific factors. These contribute to the complexity of DVT epidemiology on the continent and often complicate prevention and management efforts. Understanding these risk factors is crucial for tailoring effective public health interventions in line with the WHO Africa 2030 agenda (Table 1) (23). Epidemiological datasets (e.g., Global Burden of Disease) quantify incidence, prevalence, burden, and risk factors across conditions, while real-world evidence (RWE) uses routine clinical data to understand how diseases and treatments behave in everyday practice. Large GBD analyses provide standardized incidence, prevalence, mortality, and disability-adjusted life years (DALYs) for many diseases across 204 countries over three decades. Alzheimer's/dementias incidence and prevalence rose ∼150%–160% from 1990 to 2019; asthma incidence is projected to remain high to 2050; ischemic heart disease (IHD) prevalence is rising despite declining mortality/DALYs. Pregnancy loss and several maternal/child risks show falling age-standardized rates, though absolute numbers can rise in some countries. Major global risk contributors include air pollution, high blood pressure, smoking, high BMI and fasting glucose (24, 25). Many burdens correlate with ageing and development level: higher social development index (SDI) often means higher incidence/prevalence but lower mortality/DALYs (e.g., asthma, dementia, IHD).

**Table 1:** Risk factors unique to Africa.

Risk Factor Category	Description	Impact on DVT Risk
Infectious Diseases	High prevalence of HIV/AIDS, tuberculosis, and malaria causing chronic inflammation and endothelial dysfunction	Increased hypercoagulability and immune activation, raising DVT risk
Genetic and Hematologic	Sickle cell disease (SCD) and possible inherited thrombophilias prevalent in African populations	Hemolysis and vascular injury promote thrombosis
Healthcare Access Issues	Limited thromboprophylaxis, shortage of trained personnel, inadequate diagnostic tools	Delayed diagnosis and insufficient prevention increase DVT incidence
Sociocultural and Economic	Traditional beliefs, stigma, poverty, transportation challenges	Delayed care-seeking and poor treatment adherence
Lifestyle and Demographic	Urbanization-related obesity, sedentary lifestyle, aging population, increasing cancer and surgical rates	Amplifies classical DVT risk factors

Sex differences are common: women show higher insomnia and dementia burden, men higher IHD and COPD. Lifestyle risks (smoking, obesity, sedentary behavior, low education) are repeatedly linked to higher burden, e.g., pregnancy loss, asthma, dementia, osteoarthritis (26, 27). RWE comes from electronic health records, claims, registries, and routine data, often via observational designs or pragmatic trials.

Strengths: large, diverse populations; long-term safety/effectiveness; rare events; utilization and economic outcomes (28, 29). Key methodological challenges include confounding, selection and information bias, misclassification, missing data, coding/operationalization problems, and data quality. Advanced methods (propensity scores, target-trial emulation) and transparent reporting are emphasized to improve validity. Large epidemiological programs quantify global patterns in disease incidence, prevalence, and burden, and consistently highlight modifiable risks such as smoking, obesity, and air pollution. RWE from routine clinical data complements these estimates by showing how diseases and treatments behave in everyday practice, but it requires careful design and bias control to yield reliable conclusions (30, 31).

## Infectious diseases and coagulopathy

Infectious diseases play a significant role in predisposing African populations to DVT. HIV infection, highly prevalent in sub-Saharan Africa, is strongly associated with an increased risk of venous thromboembolism due to chronic immune activation, endothelial dysfunction, and coagulation abnormalities. The risk is further heightened by opportunistic infections, antiretroviral therapy side effects, and associated comorbidities. Similarly, tuberculosis, a common co-infection in HIV-positive patients, triggers systemic inflammation that enhances thrombosis risk. Malaria, prevalent in many regions, can also induce a hypercoagulable state through endothelial damage and platelet activation (32, 33).

## Genetic and hematologic factors

Sickle cell disease (SCD), an inherited hemoglobinopathy endemic in parts of Africa, notably contributes to thrombosis risk through mechanisms such as hemolysis, chronic inflammation, and vascular endothelial injury. Patients with SCD have an increased propensity for venous and arterial thrombosis, often compounded by repeated vaso-occlusive crises and hospitalization. Additionally, certain inherited thrombophilias, though under-researched in African populations, may play a role but remain largely undiagnosed due to lack of laboratory capacity (33).

## Healthcare access and systemic challenges

Limited access to quality healthcare services significantly impacts DVT risk. Many healthcare facilities lack standardized thromboprophylaxis protocols, particularly in perioperative and obstetric care, leading to missed opportunities for prevention. Surgical and trauma patients often do not receive anticoagulant prophylaxis due to cost, supply issues, or lack of awareness among healthcare workers. Similarly, prolonged immobilization due to delayed hospital admissions or inadequate rehabilitation services increases susceptibility to venous thrombosis (34).

## Sociocultural practices and economic barriers

Cultural beliefs and practices influence health-seeking behaviors, which may delay diagnosis and treatment of DVT. For instance, reliance on traditional medicine or stigma associated with chronic diseases can prevent timely access to medical care. Economic constraints, including the high cost of diagnostic imaging and anticoagulant medications, restrict patients’ ability to obtain essential interventions. Transportation challenges in rural areas further limit consistent follow-up and adherence to treatment (35).

## Lifestyle and demographic changes

Rapid urbanization and lifestyle transitions across Africa have led to an increase in obesity, sedentary behavior, and other metabolic risk factors linked to DVT. Pregnancy and the postpartum period, already established risk factors for venous thromboembolism, pose additional challenges in settings with limited maternal health services and insufficient thromboprophylaxis. Aging populations and increasing prevalence of malignancies also contribute to the rising DVT burden (36).

## WHO's agenda for Africa 2030: strategic priorities

The World Health Organization (WHO) has recognized deep vein thrombosis (DVT) and venous thromboembolism (VTE) as growing public health concerns in Africa and has incorporated their prevention and management into its comprehensive health agenda for the continent through 2030. This agenda aligns with the Sustainable Development Goals (SDGs) and the WHO Global Action Plan for the Prevention and Control of Non-Communicable Diseases, emphasizing the need to reduce premature mortality from cardiovascular diseases and improve overall vascular health. The WHO's strategic priorities for DVT in Africa focus on strengthening health systems, enhancing surveillance, and promoting equitable access to care (Table 2) (37, 38).

**Table 2:** WHO's agenda for Africa 2030: strategic priorities.

Strategic Priority	Key Focus Areas	Intended Outcomes
Surveillance and Data Systems	Establish national registries, integrate DVT data into health information systems	Improved epidemiological understanding and resource allocation
Capacity Building	Training healthcare workers, developing thrombosis management skills	Enhanced early detection and effective treatment
Access to Diagnostics and Treatment	Expand availability of Doppler ultrasound, affordable anticoagulants	Timely diagnosis and improved therapeutic outcomes
Integration into Existing Programs	Embed DVT prevention in maternal health, HIV/AIDS, surgical care	Holistic care and optimized resource utilization
Community Awareness and Education	Public campaigns, engagement with community leaders and traditional healers	Increased awareness, early healthcare seeking, and adherence
Research and Innovation	Support context-specific studies, promote point-of-care diagnostics, telemedicine	Evidence-based interventions and overcoming logistical barriers

## Enhancing surveillance and data systems

A cornerstone of the WHO agenda is the establishment of robust epidemiological surveillance systems across African countries. Accurate, timely data on the incidence, prevalence, and outcomes of DVT are critical for evidence-based policy formulation and resource allocation. The WHO promotes the integration of DVT indicators into national health information systems and encourages the development of registries and population-based studies. Enhanced surveillance will help identify high-risk populations and monitor the effectiveness of interventions over time (39).

## Capacity building and workforce training

Addressing the shortage of trained healthcare professionals proficient in diagnosing and managing DVT is a key priority. The WHO supports capacity-building initiatives that provide training for clinicians, nurses, and allied health workers in recognizing risk factors, utilizing diagnostic tools such as Doppler ultrasound, and implementing thromboprophylaxis protocols. Strengthening multidisciplinary teams within hospitals and community settings ensures timely, effective care and reduces complications from delayed diagnosis (40).

## Improving access to diagnostics and treatment

Limited availability of diagnostic tools and anticoagulant medications hampers effective DVT management in many African settings. The WHO agenda emphasizes the need to improve infrastructure, including expanding access to affordable Doppler ultrasonography and laboratory coagulation testing. Additionally, ensuring the availability and affordability of essential medicines, such as low-molecular-weight heparins and oral anticoagulants, is fundamental to reducing mortality and morbidity. Policies encouraging the rational use of anticoagulants and monitoring treatment adherence are also promoted (41).

## Integration into existing health programs

To optimize resource use, the WHO advocates for integrating DVT prevention and management into existing health programs, especially those targeting cardiovascular diseases, maternal health, HIV/AIDS, and surgical care. For instance, routine thromboprophylaxis can be incorporated into perioperative protocols, obstetric care, and HIV clinics. This integrated approach facilitates comprehensive patient management and leverages existing healthcare infrastructure (42).

## Community awareness and education

Raising public awareness about DVT risk factors, signs, and the importance of early medical intervention is vital. The WHO encourages culturally sensitive education campaigns targeting communities and healthcare workers to dispel myths and promote preventive behaviors, such as early mobilization after surgery and adherence to treatment regimens. Engaging community leaders and leveraging media platforms are key strategies in these efforts (43).

## Research and innovation

Finally, the WHO agenda supports fostering research tailored to the African context to address knowledge gaps related to DVT pathophysiology, risk factors, and optimal interventions. Innovation in point-of-care diagnostics, telemedicine, and affordable anticoagulant therapies is encouraged to overcome logistical and economic barriers. Collaborative partnerships between governments, academic institutions, and international organizations are essential for advancing this research agenda (44, 45).

## Prognostic stratification and clinical outcomes in deep vein thrombosis

Prognostic stratification has become an increasingly important component of DVT and VTE management, particularly in healthcare systems with limited resources where early identification of high-risk patients may significantly improve outcomes. Although traditional risk assessment has largely focused on the presence of provoking factors such as surgery, immobilization, malignancy, trauma, pregnancy, and hospitalization, emerging evidence indicates that baseline clinical and laboratory parameters play critical roles in determining disease severity, recurrence risk, therapeutic response, and mortality outcomes (46, 47). Clinical outcomes in DVT vary substantially depending on patient characteristics, comorbidities, timeliness of diagnosis, and access to anticoagulation therapy. While many patients recover without major complications following appropriate treatment, others develop severe outcomes including pulmonary embolism, recurrent thrombosis, post-thrombotic syndrome, chronic venous insufficiency, chronic thromboembolic pulmonary hypertension, and death. In African healthcare settings, delayed presentation, diagnostic limitations, and restricted access to specialized thrombosis care may further worsen prognosis (48, 49).

Recent real-world studies and registry-based analyses have emphasized the prognostic significance of simple baseline laboratory variables that may be readily applicable in low-resource settings. Hemoglobin levels, for example, have emerged as important predictors of adverse clinical outcomes in VTE patients. Anemia has been associated with increased mortality, recurrent thromboembolic events, prolonged hospitalization, and greater bleeding risk during anticoagulation therapy. Reduced hemoglobin levels may reflect underlying chronic disease, occult malignancy, malnutrition, renal dysfunction, inflammatory states, or advanced disease severity, all of which contribute to poorer prognosis. The incorporation of hemoglobin assessment into routine clinical evaluation may therefore provide an accessible and cost-effective prognostic tool in resource-constrained African health systems (50, 51). In addition to hemoglobin, several other laboratory and clinical markers have demonstrated prognostic value. Elevated inflammatory markers, impaired renal function, thrombocytopenia, advanced age, obesity, active malignancy, heart failure, and concomitant infectious diseases such as HIV and tuberculosis have all been linked to worse clinical outcomes in patients with DVT. Persistent inflammatory activation and endothelial dysfunction may further contribute to recurrent thrombosis and long-term vascular complications (52, 53).

The coexistence of infectious and non-communicable diseases in Africa presents unique prognostic challenges. HIV-associated thrombosis is frequently characterized by advanced immune activation, opportunistic infections, malignancy, and treatment-related metabolic disturbances, all of which may increase thrombotic burden and complicate anticoagulation management. Similarly, tuberculosis-associated thrombosis often occurs in severely ill patients with extensive inflammatory responses and prolonged immobilization, thereby increasing mortality risk (54). Risk stratification is particularly important in predicting the development of pulmonary embolism, the most life-threatening complication of DVT. Patients presenting with hemodynamic instability, respiratory compromise, extensive proximal thrombosis, or significant comorbid conditions require urgent intervention and closer monitoring. Early identification of high-risk patients can facilitate timely escalation of care, improve allocation of limited healthcare resources, and reduce preventable mortality (55, 56).

Long-term clinical outcomes also remain a major concern. Post-thrombotic syndrome, characterized by chronic pain, edema, skin changes, and venous ulceration, significantly impairs quality of life and contributes to disability and economic hardship. Recurrent thrombosis further increases healthcare utilization and long-term morbidity. In many African countries where rehabilitation services and chronic vascular care are limited, these complications may have profound socioeconomic consequences (57). The growing availability of real-world registry data has improved understanding of factors influencing VTE outcomes. Large observational studies have demonstrated that clinical outcomes are affected not only by anticoagulation therapy but also by comorbidity burden, healthcare access, treatment adherence, nutritional status, and concomitant medications. Emerging evidence has additionally suggested that therapies such as statins may exert protective anti-inflammatory and endothelial-stabilizing effects that could potentially influence mortality outcomes in VTE patients, although further studies are required to establish definitive clinical benefit in African populations (58, 59).

## Challenges and barriers

Despite the WHO's strategic priorities and growing recognition of deep vein thrombosis (DVT) as a public health concern in Africa, numerous challenges impede effective prevention, diagnosis, and management across the continent. These barriers are multifaceted, encompassing health system limitations, socioeconomic factors, and cultural influences that collectively hinder progress toward WHO's 2030 goals (60).

## Health system limitations

Many African countries face systemic healthcare constraints, including inadequate infrastructure, shortage of trained personnel, and limited availability of diagnostic tools necessary for timely DVT identification. Doppler ultrasound machines, the gold standard for DVT diagnosis, are often scarce, particularly in rural and resource-limited settings. Laboratory facilities capable of performing coagulation tests are similarly lacking or inconsistently available. This scarcity results in delayed or missed diagnoses, contributing to increased morbidity and mortality. Additionally, fragmented referral systems and poor coordination among primary, secondary, and tertiary care facilities further complicate patient management (43, 61).

## Limited access to anticoagulant therapy

Access to essential anticoagulant medications such as low-molecular-weight heparins and direct oral anticoagulants (DOACs) remains a significant barrier. Many patients cannot afford these drugs, and inconsistent supply chains result in frequent stockouts in public health facilities. Even where medications are available, the lack of monitoring capabilities for anticoagulation therapy (e.g., INR testing for warfarin) and inadequate patient follow-up compromise treatment safety and efficacy. These factors often lead to suboptimal adherence, increased risk of complications, and recurrent thrombotic events (62).

## Sociocultural and economic barriers

Cultural beliefs and misconceptions about blood disorders and chronic diseases can deter patients from seeking prompt medical attention or adhering to prescribed treatments. In some communities, symptoms of DVT may be attributed to supernatural causes or traditional illnesses, leading patients to consult traditional healers instead of biomedical facilities. Economic hardships, including poverty and transportation challenges, limit access to healthcare services and diagnostic testing, especially for rural populations. Out-of-pocket costs for consultations, investigations, and long-term therapy often create prohibitive financial burdens (63).

## Low awareness and education Among healthcare workers and the public

Limited awareness and knowledge of DVT risk factors, clinical presentation, and management guidelines among healthcare workers contribute to underdiagnosis and inappropriate treatment. This knowledge gap is compounded by the absence of standardized training curricula and continuing medical education focused on thrombosis. Public awareness is equally low, with many individuals unaware of DVT symptoms or the importance of preventive measures such as early mobilization after surgery or during prolonged illness (64).

## Competing health priorities

Infectious diseases such as HIV/AIDS, tuberculosis, and malaria, as well as emerging non-communicable diseases like hypertension and diabetes frequently overwhelm African health systems. The prioritization of these conditions, coupled with constrained resources, often sidelines thrombosis prevention and management programs. Additionally, policy frameworks and funding streams dedicated explicitly to DVT and venous thromboembolism are limited, resulting in insufficient programmatic support (65).

## Data deficiency and research gaps

The absence of comprehensive epidemiological data on DVT in Africa hinders advocacy efforts and policy development. Limited research capacity and funding restrict studies on local risk factors, diagnostic innovations, and context-appropriate interventions. This knowledge gap perpetuates a cycle of under-recognition and under-resourcing of DVT programs (66).

## Management and therapeutic implications of deep vein thrombosis in Africa

The management of DVT aims to prevent thrombus propagation, pulmonary embolism, recurrent VTE, post-thrombotic syndrome, and mortality while minimizing treatment-related complications, particularly bleeding. In Africa, however, optimal management of DVT remains constrained by multiple health system limitations including delayed diagnosis, inadequate access to anticoagulants, insufficient laboratory monitoring, workforce shortages, and financial barriers to care. These challenges significantly influence therapeutic outcomes and contribute to preventable morbidity and mortality across the continent (44, 45). Anticoagulation remains the cornerstone of DVT treatment. Conventional therapeutic approaches include unfractionated heparin, low-molecular-weight heparin (LMWH), and vitamin K antagonists such as warfarin. More recently, direct oral anticoagulants (DOACs) have transformed thrombosis management globally due to their predictable pharmacokinetics, reduced monitoring requirements, and improved convenience. However, access to DOACs remains limited in many African countries because of high costs, inconsistent drug supply chains, and limited insurance coverage (46).

The choice of anticoagulant in African healthcare settings is often influenced more by availability and affordability than by individualized clinical considerations. In many low-resource hospitals, unfractionated heparin and warfarin remain the most accessible options despite the need for regular coagulation monitoring and dose adjustment. Unfortunately, monitoring systems such as international normalized ratio (INR) testing are inconsistently available, especially in rural and underserved communities, thereby increasing the risk of subtherapeutic anticoagulation, recurrent thrombosis, or bleeding complications (47, 48). Several factors influence outcomes during anticoagulation therapy among DVT patients in Africa. Delayed presentation to healthcare facilities frequently results in advanced disease severity at the time of diagnosis, including extensive proximal thrombosis and pulmonary embolism. Inadequate awareness of DVT symptoms among both healthcare providers and the public contributes to diagnostic delays and missed opportunities for early intervention (49).

Medication adherence also significantly affects therapeutic outcomes. Long treatment durations, financial hardship, transportation challenges, and medication shortages may reduce adherence to anticoagulant therapy. Poor adherence increases the risk of recurrent VTE, chronic complications, and mortality. Patient education regarding the importance of treatment continuity, medication interactions, and recognition of bleeding symptoms is therefore essential (50). Bleeding risk remains a major concern during anticoagulation therapy, particularly among patients with comorbid conditions such as liver disease, renal impairment, severe anemia, malnutrition, cancer, and infectious diseases. Resource-limited healthcare settings may face additional challenges in managing anticoagulation-related bleeding due to limited blood product availability, inadequate emergency services, and restricted access to reversal agents (51). Recent evidence from real-world studies has demonstrated that baseline clinical and laboratory parameters may influence outcomes during anticoagulation therapy. Factors such as low hemoglobin levels, renal dysfunction, advanced age, active malignancy, inflammatory burden, and multiple comorbidities have been associated with poorer prognosis and increased mortality among VTE patients. These findings are particularly relevant in African settings where advanced diagnostic tools may be limited and accessible prognostic markers can assist clinical decision-making (52).

The coexistence of infectious diseases further complicates DVT management in Africa. HIV infection may alter coagulation pathways, increase drug-drug interactions, and influence anticoagulant metabolism, particularly among patients receiving antiretroviral therapy. Tuberculosis treatment similarly presents challenges due to interactions between rifampicin and oral anticoagulants, potentially reducing therapeutic efficacy. These complexities necessitate careful monitoring and individualized treatment strategies (53). Cancer-associated thrombosis presents additional therapeutic difficulties. Patients with malignancy are at increased risk of both thrombosis recurrence and anticoagulation-related bleeding. Limited oncology infrastructure and delayed cancer diagnosis in many African countries further worsen outcomes. Expanding multidisciplinary collaboration between hematologists, oncologists, surgeons, and primary care providers is essential for improving care in these high-risk populations (54). Emerging evidence also suggests that concomitant therapies may influence clinical outcomes in VTE patients. Registry-based analyses have explored the potential role of statins in reducing mortality and improving vascular outcomes due to their anti-inflammatory, endothelial-protective, and antithrombotic effects. Although further evidence is needed, such findings highlight the importance of comprehensive cardiovascular risk management in thrombosis care (55).

Beyond pharmacological treatment, supportive and preventive interventions remain critical components of DVT management. Early mobilization, hydration, mechanical thromboprophylaxis, compression therapy, and lifestyle modification may reduce complications and improve long-term outcomes. Hospital-based thromboprophylaxis programs are particularly important for surgical patients, trauma patients, critically ill individuals, and pregnant women at elevated thrombotic risk (56). Health systems strengthening is central to improving therapeutic outcomes for DVT in Africa. Expanding access to affordable anticoagulants, strengthening laboratory infrastructure, improving healthcare worker training, developing standardized treatment protocols, and integrating thrombosis care into primary healthcare systems are essential priorities. Telemedicine and digital health technologies may also improve anticoagulation monitoring and follow-up care in underserved regions (57, 58).

## Future research priorities for Africa

Despite growing recognition of DVT and VTE as important contributors to morbidity and mortality in Africa, substantial knowledge gaps continue to limit evidence-based policymaking, resource allocation, and clinical decision-making. The available literature remains fragmented, with most data originating from single-center studies, tertiary hospitals, and urban healthcare facilities. Consequently, a coordinated research agenda is required to generate robust evidence capable of guiding thrombosis prevention and management strategies across diverse African healthcare settings (49). One of the most urgent priorities is the establishment of national and regional VTE registries. The absence of comprehensive surveillance systems has contributed to significant underestimation of the true burden of thrombosis across the continent. Well-designed registries would facilitate accurate assessment of disease incidence, prevalence, risk factors, treatment patterns, clinical outcomes, and mortality. Such data are essential for informing national health policies, evaluating healthcare interventions, and monitoring progress toward public health goals aligned with the WHO Agenda for Africa 2030 (50, 51).

Population-based epidemiological studies are also needed to address existing gaps in knowledge regarding the distribution and determinants of DVT among African populations. Current estimates are largely derived from hospital-based cohorts, which may not adequately capture the burden of disease in rural communities and underserved populations. Large multicenter studies involving diverse geographical regions would improve understanding of regional variations in thrombosis risk and disease presentation while identifying context-specific determinants of adverse outcomes (52). Given the significant disparities in healthcare access across the continent, future research should prioritize validation of diagnostic strategies suitable for resource-limited environments. Rural and community-based studies evaluating simplified clinical prediction tools, point-of-care D-dimer testing, portable ultrasonography, and telemedicine-supported diagnostic approaches could help improve early detection of DVT in areas where advanced imaging facilities are unavailable. Such investigations would be particularly valuable for strengthening primary healthcare systems and expanding equitable access to thrombosis care (53).

Further research is required to better understand the unique interactions between infectious diseases and thrombosis in Africa. HIV infection, tuberculosis, malaria, viral infections, and other endemic diseases may influence coagulation pathways through mechanisms involving chronic inflammation, endothelial dysfunction, immune activation, and thromboinflammation. Although these associations are, increasingly recognized, important questions remain regarding disease-specific risk profiles, optimal thromboprophylaxis strategies, anticoagulation management, and long-term outcomes in affected populations (54). Another critical research priority involves the development and validation of African-specific prognostic and risk stratification models. Most currently available prediction tools have been developed in high-income settings and may not adequately account for the epidemiological, socioeconomic, and healthcare characteristics unique to African populations. Studies incorporating locally relevant variables, including infectious disease burden, healthcare accessibility, nutritional status, and demographic factors, could facilitate more accurate identification of high-risk patients and support individualized management strategies (55).

.Pragmatic clinical trials are also needed to evaluate the effectiveness and feasibility of thrombosis prevention interventions within real-world African healthcare environments. Research should assess context-specific approaches to thromboprophylaxis in hospitalized patients, surgical populations, pregnant women, cancer patients, and individuals living with HIV or tuberculosis. Such studies would provide critical evidence regarding intervention effectiveness, safety, implementation barriers, and cost-effectiveness under routine clinical conditions (56). Implementation science represents another important area for future investigation. Although international guidelines for thrombosis prevention and management are widely available, their adoption remains inconsistent across many African healthcare institutions. Research exploring barriers to guideline implementation, healthcare worker training needs, institutional practices, and patient-level factors could inform strategies aimed at improving adherence to evidence-based care and enhancing clinical outcomes (57). The growing availability of digital health technologies presents additional opportunities for innovation in thrombosis care. Future studies should evaluate the role of telemedicine platforms, mobile health applications, electronic clinical decision-support systems, and artificial intelligence-assisted risk prediction tools in improving thrombosis screening, diagnosis, treatment monitoring, and patient follow-up. These technologies may offer scalable solutions for addressing healthcare workforce shortages and geographic barriers to care.

Economic evaluations are equally important. Cost-effectiveness analyses examining thromboprophylaxis programs, diagnostic technologies, anticoagulation strategies, and health system interventions are necessary to support efficient allocation of limited healthcare resources. Such evidence will be particularly valuable for policymakers seeking to integrate thrombosis prevention into broader universal health coverage and non-communicable disease frameworks (57). Strengthening regional and international research collaborations should be considered a strategic priority. Multicenter networks involving academic institutions, professional societies, ministries of health, and international organizations can facilitate data sharing, capacity building, harmonization of research methodologies, and development of continent-wide thrombosis research initiatives. These collaborations have the potential to accelerate scientific discovery and support the generation of high-quality evidence tailored to African healthcare needs.

## Recommendations

To effectively address the growing burden of deep vein thrombosis (DVT) in Africa and realize the goals of the WHO's 2030 agenda, a comprehensive, context-specific, and multisectoral strategy is imperative. The following recommendations target key areas including healthcare system strengthening, community engagement, policy development, and research.

## Strengthening healthcare infrastructure and capacity

Investment in healthcare infrastructure is essential to improve diagnostic and treatment capabilities for DVT. This includes expanding access to Doppler ultrasound and laboratory coagulation testing at secondary and tertiary care levels, particularly in underserved rural regions. Training and continuous professional development programs should be prioritized for healthcare workers, focusing on early recognition, risk assessment, and management of DVT using locally adapted guidelines. Establishing multidisciplinary teams comprising physicians, nurses, and allied health professionals will facilitate coordinated care and improve patient outcomes.

## Improving access and affordability of anticoagulant therapies

Governments and stakeholders should work toward ensuring a stable supply of affordable anticoagulant medications, including low-molecular-weight heparins and direct oral anticoagulants (DOACs). National procurement and supply chain systems must be strengthened to reduce stockouts and price fluctuations. Implementation of task-shifting models can support decentralized anticoagulation monitoring, enabling community health workers to assist with follow-up and adherence support, especially in resource-limited settings.

## Integrating DVT prevention into existing health programs

DVT prevention and management should be incorporated into existing public health initiatives such as maternal health, HIV/AIDS, surgical care, and non-communicable disease programs. For example, routine thromboprophylaxis protocols can be embedded into perioperative and antenatal care pathways. Integration optimizes resource utilization, enhances patient coverage, and supports holistic care approaches for individuals with multiple comorbidities.

## Enhancing public awareness and community education

Culturally sensitive education campaigns are needed to raise awareness of DVT risk factors, symptoms, and the importance of early medical intervention. Community leaders, traditional healers, and media outlets should be engaged to disseminate accurate information and dispel misconceptions. Educational initiatives must also emphasize preventive measures such as early mobilization, hydration, and compliance with prescribed treatments.

## Policy development and advocacy

National health policies should explicitly include DVT and venous thromboembolism within non-communicable disease and cardiovascular health frameworks. Policymakers must allocate sufficient funding for thrombosis-related programs and encourage the development of national clinical guidelines that reflect local epidemiology and resource availability. Advocacy efforts at regional and international levels can mobilize technical and financial support to sustain these initiatives.

## Promoting research and data collection

Expanding research capacity in Africa is critical to generate context-specific evidence on DVT epidemiology, risk factors, and effective interventions. Collaborative partnerships among academic institutions, healthcare providers, and governments should be fostered to conduct population-based studies and clinical trials. Investment in surveillance systems and national registries will provide the data necessary to monitor trends, evaluate program impact, and inform continuous improvement.

## Leveraging technology and innovation

The adoption of innovative technologies such as point-of-care diagnostics, telemedicine, and mobile health platforms can overcome geographical and resource barriers. Digital tools can support remote diagnosis, patient monitoring, and education, particularly in rural areas with limited access to specialized care.

## Conclusion

DVT poses a significant and growing public health challenge across Africa, driven by a complex interplay of infectious diseases, genetic predispositions, emerging non-communicable risk factors, and systemic healthcare limitations. Despite the burden, DVT remains under-recognized and underdiagnosed due to inadequate surveillance, limited diagnostic capacity, and low awareness among healthcare providers and the public. The WHO's Agenda for Africa 2030 provides a strategic framework to address these challenges through strengthened health systems, capacity building, improved access to diagnostics and treatment, and integrated public health programming. To translate this agenda into measurable health gains, concerted efforts are required at multiple levels — from policy formulation and healthcare infrastructure investment to community education and research innovation. Addressing sociocultural and economic barriers alongside systemic health challenges is essential to ensure equitable access to preventive and therapeutic services. Furthermore, fostering robust data collection and context-specific research will fill critical knowledge gaps and guide effective interventions.
